# Comparing Treatment Acceptability and 12-Month Cessation Rates in Response to Web-Based Smoking Interventions Among Smokers Who Do and Do Not Screen Positive for Affective Disorders: Secondary Analysis

**DOI:** 10.2196/13500

**Published:** 2019-06-19

**Authors:** Noreen L Watson, Jaimee L Heffner, Kristin E Mull, Jennifer B McClure, Jonathan B Bricker

**Affiliations:** 1 Fred Hutchinson Cancer Research Center Seattle, WA United States; 2 Kaiser Permanente Washington Health Research Institute Seattle, WA United States; 3 University of Washington Seattle, WA United States

**Keywords:** smoking, smoking cessation, affective disorders, anxiety, depression, eHealth, Web intervention, co-occurring disorders

## Abstract

**Background:**

Web-based cessation programs are now common for intervening with smokers. However, it remains unclear how acceptable or effective these interventions are among people with affective disorders and symptoms (ADS; eg, depression and anxiety). Research examining this is extremely limited, with mixed results on cessation rates. Additional large studies are needed to more fully understand whether Web-based interventions are similarly used and equally effective among people with and without affective disorder symptomology. If not, more targeted Web-based interventions may be required.

**Objective:**

The goal of the research was to compare Web-based treatment acceptability (defined by satisfaction and use) and 12-month cessation outcomes between smokers with and without ADS.

**Methods:**

Participants (N=2512) were adult smokers enrolled in a randomized, comparative effectiveness trial of two Web-based smoking interventions designed for the general population of smokers. At baseline, participants reported demographic and smoking characteristics and completed measures assessing ADS. Participants were then classified into subgroups based on their self-reported ADS—either into a no ADS group or into six nonmutually exclusive subgroups: depression, posttraumatic stress disorder (PTSD), panic disorder (PD), generalized anxiety disorder (GAD), social anxiety disorder (SAD), and more than one ADS. Surveys at 12 months postrandomization included subjective ratings of treatment acceptability and self-reported smoking cessation. Treatment use (ie, number of log-ins and total duration of exposure) was assessed via automated records.

**Results:**

Relative to the no ADS group, all six ADS subgroups reported significantly greater satisfaction with their assigned Web treatment program, but they spent less time logged in than those with no ADS. For number of log-ins, a treatment arm by ADS group interaction was observed across all ADS subgroups except GAD, suggesting that relative to the no ADS group, they logged in less to one website but not the other. At the 12-month follow-up, abstinence rates in the no ADS group (153/520, 29.42%) were significantly higher than for participants who screened positive for depression (306/1267, 24.15%; *P*=.03), PTSD (294/1215, 24.19%; *P*=.03), PD (229/1003, 23.83%; *P*=.009), and two or more ADS (323/1332, 24.25%; *P*=.03). Post hoc analyses suggest the lower quit rates may be associated with differences in baseline nicotine dependence and levels of commitment to resist smoking in difficult situations. Website use did not explain the differential abstinence rates.

**Conclusions:**

Despite reporting higher levels of treatment satisfaction, most smokers with ADS used their assigned intervention less often and had lower quit rates than smokers with no ADS at treatment onset. The results support the need for developing more targeted interventions for smokers with ADS.

**Trial registration:**

Clinical Trials.gov NCT01812278; https://clinicaltrials.gov/ct2/show/NCT01812278 (Archived by WebCite at http://www.webcitation.org/78L9cNdG4)

## Introduction

With lifetime prevalence rates ranging from 16.6% to 28.8%, affective disorders such as depression and anxiety are among the most common mental health conditions in the United States [1]. Epidemiological data indicate that individuals with these disorders, as well as those with elevated symptoms of anxiety or depression that do not meet a diagnostic threshold, have significantly higher prevalence rates of smoking and are less likely to successfully quit than those without such conditions or symptoms [2-6]. As a result, smokers with affective disorders and symptoms (ADS) account for disproportionate rates of tobacco-related deaths and diseases [7,8], and ongoing efforts are needed to improve cessation rates among this at-risk group of smokers.

One way to increase rates of cessation among smokers with ADS is to make treatment more accessible. This can be accomplished with interventions that are low-burden, low-cost, and high-reach, including Web-based interventions. Indeed, each year an estimated 12 million smokers look online for help to quit smoking [9]. In the general population of smokers, Web-based interventions demonstrated similar levels of effectiveness as more traditional treatment modalities such as face-to-face and telephone-delivered interventions in a recent meta-analysis [10]. However, there is a paucity of research examining the acceptability and effectiveness of these nontargeted interventions for individuals exhibiting ADS, who may have unique needs not addressed in Web-based interventions designed for the general population. Results from the few Web-based intervention studies reporting smoking cessation outcomes for individuals with ADS have yielded mixed findings with regard to their effectiveness for cessation. Of the two studies that examined anxiety, one demonstrated that individuals with greater levels of anxiety were less likely to quit and more likely to relapse compared with their less anxious counterparts at 4-week follow-up [11], whereas the other did not find a relationship between anxiety and quitting smoking at 3-month follow-up [12]. In addition, neither study examined how anxiety influenced long-term quit rates, both studies had low rates of outcome data retention, and both studies used broad measures of anxiety rather than comparing outcomes among different types of anxiety disorders or symptom categories. Thus, additional research addressing these limitations is needed. With regard to the impact of depression on smoking cessation when using Web-based interventions, two studies have demonstrated that 13-month quit rates were significantly lower among individuals with current depressive symptoms versus those with no depression [13,14]. However, three studies have demonstrated that current depressive symptoms were not associated with smoking cessation at 3 months [12,15] or 12 months [16]. In short, it remains unclear whether standard Web-based smoking interventions (ie, those not targeted for special populations) are less effective for individuals with ADS.

Based on research with other (non-Web) treatment modalities, one might expect that standard Web-based interventions would be less effective for smokers with ADS relative to those who do not have this symptomology [5,17-20] or at least less effective for some types of ADS. For example, because individuals with social anxiety disorder often avoid interactions with others due to fears about being negatively evaluated, socially anxious smokers may respond well to Web-based treatments, as they would provide access to an evidence-based intervention without needing to interact with other smokers or providers directly. On the other hand, smokers with affective disorders associated with various degrees of cognitive impairment (eg, poor concentration), such as depression or posttraumatic stress disorder (PTSD), may not respond as well to self-guided, stand-alone Web-based interventions. Examining such disorder-specific differentiations in smoking outcomes remains unexplored in the literature, but having a greater understanding how specific ADS and comorbid affective symptomology are associated with treatment satisfaction, use, and outcome could enhance future efforts to develop more effective interventions for these.

To best guide these targeted treatment development efforts, it is important to understand how smokers with different ADS respond differentially to Web-based cessation programs relative to smokers without ADS. Thus, the primary goal of this study was to compare treatment acceptability, defined both by self-reported satisfaction and treatment use, and 12-month abstinence rates among smokers with and without symptoms of one or more common affective disorders (depression, social anxiety disorder [SAD], generalized anxiety disorder [GAD], panic disorder [PD], and PTSD). All participants were recruited as part of a prior comparative effectiveness trial of two online interventions, each targeting the general population of smokers and testing two different Web-based interventions [21]. This paper is a secondary analysis of data collected from this randomized controlled trial (RCT). We hypothesized that, regardless of the assigned Web-based treatment program, smokers with ADS at treatment onset would have less satisfaction, lower levels of engagement, and lower cessation rates than smokers with no ADS and aimed to determine if there is evidence of differential outcomes that are disorder-specific.

## Methods

### Overview

A comprehensive description of study methods can be found in the main outcomes paper [21]. In brief, participants were 2512 of the 2637 adult smokers from all 50 states enrolled in the parent RCT, which compared the effectiveness of two Web-based smoking cessation programs [NCT01812278]. For the purposes of these analyses, the analytic sample was limited to participants who had complete data on at least one screening measure assessing affective disorder symptomology (see below for more details on these measures). To be eligible for the parent trial, participants had to (1) be 18 years of age or older; (2) smoke 5 or more cigarettes per day for the last year; (3) express a desire to quit smoking within 30 days; (4) have access to high-speed internet, including access to email; (5) not be participating in other smoking interventions or treatments; (6) never have used the control intervention, Smokefree.gov; (7) never have participated in one of our previous smoking studies; (8) not have another member in their household participating in the study; (9) express a willingness to be randomized to treatment, complete 3 follow-up surveys, and provide contact information for themselves and two relatives; (10) reside in the United States; and (11) be able to read in English. After completing the informed consent and baseline survey online, participants were randomized to one of two Web-based programs based either on acceptance and commitment therapy (ACT) or standard care (the National Cancer Institute’s Smokefree.gov website). The ACT-based website presented content in a sequential manner through four phases and prompted users to track their smoking each day. The Smokefree.gov website had three main sections and users could navigate through all pages of the website freely with no restrictions on the order in which content had to be viewed. Participants had access to their assigned interventions for 12 months and received up to 4 messages per day for 28 days (via text or email) designed to encourage website use. Outcome surveys were completed at 3, 6, and 12 months postrandomization. The 12-month data retention rate was 88%, and neither treatment arm [21] nor mental health status predicted retention [22]. Further details on the recruitment and retention methods can be found elsewhere [22].

### Measures

#### Baseline Demographics, Smoking, and Smoking-Related Characteristics

At baseline, participants reported demographic characteristics including age, gender, race and ethnicity, marital status, education, employment status, and sexual orientation. Level of nicotine dependence was assessed using the Fagerström Test for Nicotine Dependence (FTND) [23]. Scores on the FTND range from 0 to 10, with higher scores representing greater nicotine dependence. Participants also completed the Commitment to Quitting Smoking Scale (CQSS) [24] as a measure of their commitment to resist smoking in the face of difficulties, cravings, and negative affect; scores on the CQSS range from 1 to 5, with higher scores representing greater commitment to resist smoking. Smoking-specific experiential avoidance, or the tendency to smoke cigarettes in order avoid the experience of cigarette cravings, was measured with the physical sensations subscale [25] of the Avoidance and Inflexibility Scale (AIS) (adapted from Gifford et al [26]), which yields scores ranging from 1 to 5, with lower scores representing a greater avoidance of physical sensations that cue smoking. Participants were also asked to report on their use of e-cigarettes.

#### Symptoms of Depression and Anxiety Disorders

To assess for elevated levels depression and anxiety, participants completed validated self-report screening instruments for five common affective disorders. Symptoms of depression were assessed with the Center for Epidemiologic Studies Depression scale using the recommended score of ≥16 to indicate elevated symptoms [27]. Elevated symptoms of PTSD were indicated by the recommended score of ≥14 on the 6-item PTSD Checklist [28]. The Autonomic Nervous System Questionnaire was used to assess symptoms of PD; elevated PD symptoms were indicated if participants reported having ≥1 panic attacks within the past month with at least one occurring in a situation in which they were not in danger or the center of attention [29]. A score of ≥10 on the 7-Item Generalized Anxiety Disorder scale [30] indicated elevated symptoms of GAD. Finally, a score of ≥6 the Mini-Social Phobia Inventory [31] indicated elevated symptoms of SAD.

Smokers who screened positive for one or more affective disorders were classified into six nonmutually exclusive subgroups based on their baseline affective disorder symptomology: depression, PTSD, PD, GAD, SAD, and more than one affective disorder. Thus, these participants could be classified into more than one ADS subgroup. Participants who did not screen positive for any affective disorder listed above were categorized as not having elevated symptoms of any affective disorder (No AD).

#### Treatment Acceptability: Satisfaction and Use

As a subjective measure of treatment acceptability, participants answered three treatment satisfaction questions at the 3-month follow-up survey (eg, How useful did you find your assigned website?). Response options ranged from 1 (not at all) to 5 (very much), with a score ≥2 (ie, somewhat or more) indicating participant satisfaction. Additionally, website engagement over the 12-month period was calculated from data automatically logged by the secured server as indices of two objective measures of treatment acceptability—number of log-ins (primary use outcome) and total minutes spent on the website (secondary use outcome). We qualified user activity occurring more than 15 minutes after the last instance of activity as a new log-in.

#### Cessation Outcomes

Consistent with primary cessation outcomes reported in the parent trial, the primary cessation outcome for this study was self-reported 30-day point prevalence abstinence (PPA) (no smoking, not even a puff in the last 30 days) at 12 months postrandomization analyzed using a complete case methodology. The secondary cessation outcome also used 30-day PPA but with missing values imputed as smoking. Biochemical confirmation was not used in accordance with recommendations for assessing smoking status in large, population-based cessation trials in which there is no face-to-face contact and the demand characteristics for false reporting are minimal [32].

### Statistical Analysis

For descriptive purposes, baseline characteristic variables of participants with ADS (each diagnostic group considered separately) were examined and compared against the no AD group using chi-square tests for categorical variables and *t* tests for continuous variables. Logistic regression models were used to evaluate differences in treatment satisfaction and cessation outcomes between each diagnostic group and the no AD group. Negative binomial models were used to evaluate differences in website use between groups. Models included variables used in stratified randomization for the main trial (ie, gender, education, and smoking more than 20 cigarettes per day), treatment arm, and treatment arm by diagnostic group interactions were considered. Nonsignificant interaction terms were dropped from final models. Analyses were completed using R version 3.4.2 (The R Foundation) and R package MASS [33], and all statistical tests were two-sided with alpha=.05.

## Results

### Participant Symptomology

Of the 2512 participants who provided sufficient data on the screening measures to establish whether the screen was positive or negative, the majority (1938/2512, 77.15%) screened positive for at least one affective disorder at baseline. Of those, the majority screened positive for depression (1470/1938, 75.85%), followed by PTSD (1383/1938, 71.36%), PD (1145/1938, 59.08%), GAD (903/1938, 46.59%), and SAD (797/1938, 41.25%). Additionally, as can be seen in Table 1, most of these participants screened positive for more than one affective disorder. Overall, 15.18% (355/2339) screened positive for one affective disorder, 13.59% (318/2339) screened positive for two, 16.76% (392/2339) screened positive for three, 15.01% (351/2339) screened positive for four, and 14.92% (349/2339) screened positive for all five affective disorders assessed in the study, suggesting high levels of elevated affective symptomology overall.

### Baseline Characteristics

The baseline characteristics of all groups can be found in Table 2. Participants with ADS differed from those without ADS in a number of ways on demographic and smoking variables. Compared to smokers without ADS, all ADS subgroups were significantly younger, less educated, and more likely to identify as a sexual minority (ie, lesbian, gay, or bisexual). Some groups (GAD, SAD) comprised a greater proportion of women, and all ADS subgroups except the PD subgroup were less likely to be married. No differences were found between groups regarding race or ethnicity. Relative to the group without ADS, all ADS subgroups had significantly greater levels of nicotine dependence (all *P*<.001), avoidance of physical sensations that cue smoking (eg, urges) (all *P*<.001), and lower commitment to quitting scores (all *P*<.001).

### Treatment Acceptability

As can be seen in Table 3, some differences emerged regarding subjective treatment acceptability ratings and objective website use data between those with and without ADS. Contrary to our predictions, relative to those who did not screen positive for any affective disorder, all ADS subgroups reported significantly greater satisfaction with their assigned website. Although differences in ratings of the other two indices of treatment acceptability (ie, usefulness of assigned website, whether or not they would recommend the website to a friend) were not significantly different, they were in the same direction such that those with ADS reported greater acceptability. No treatment by symptom group interaction was significant.

With regard to objective website use data, our hypotheses were partially supported. Relative to the no ADS group, the GAD group logged in to their assigned website significantly fewer times (*P*<.001). For all other symptom groups, there were significant treatment by symptom group interactions such that each ADS group logged in to the Smokefree.gov website significantly fewer times than the no ADS group. No significant differences in number of log-ins between groups were found in the ACT-based arm. All symptom subgroups spent significantly less time logged in to their assigned website compared with the no ADS group, and no treatment by symptom group interaction was significant.

**Table 1:** Frequency and proportion of participants screening positive for multiple affective disorders.

ADS subgroups	Depression (n=1470), n/N (%)	PTSD^a^ (n=1383), n/N (%)	PD^b^ (n=1145), n/N (%)	GAD^c^ (n=903), n/N (%)	SAD^d^ (n=797), n/N (%)
Depression	—	1152/1378 (83.59)	854/1143 (74.72)	836/900 (92.89)	697/795 (87.67)
PTSD	1152/1450 (79.45)	—	836/1144 (73.08)	796/900 (88.44)	664/796 (82.42)
PD	854/1343 (63.59)	836/1285 (65.05)	—	597/847 (70.48)	479/733 (65.35)
GAD	836/1364 (61.29)	796/1375 (57.89)	597/1139 (52.14)	—	506/796 (63.57)
SAD	697/1468 (47.48)	664/1382 (48.05)	479/1145 (41.83)	506/902 (56.10)	—

^a^PTSD: posttraumatic stress disorder.

^b^PD: panic disorder.

^c^GAD: generalized anxiety disorder.

^d^SAD: social anxiety disorder.

**Table 2:** Baseline characteristics by affective disorder symptomology group.

Characteristics		Comparison group (no ADS^a^; n=574)	Affective disorder symptomology subgroup					
			Depression (n=1470)	PTSD^b^ (n=1383)	PD^c^ (n=1145)	GAD^d^ (n=903)	SAD^e^ (n=797)	2+ ADS^f^ (n=1529)
**Demographics**								
	Age in years, mean (SD)	49.1 (13.0)	44.5 (13.5)^g^	43.5 (13.2)^g^	43.9 (13.1)^g^	42.3 (13.4)^g^	43.3 (13.4)^g^	43.9 (13.3)^g^
	Female, n (%)	441 (76.8)	1181 (80.34)	1096 (79.25)	916 (80.00)	734 (81.28)^i^	653 (81.93)^i^	1226 (80.18)
	Caucasian, n (%)	413 (71.9)	1051 (71.49)	962 (69.56)	813 (71.00)	633 (70.09)	559 (70.14)	1079 (70.57)
	Hispanic, n (%)	43 (7.5)	134 (9.12)	137 (9.91)	107 (9.34)	96 (10.63)	80 (10.04)	148 (9.68)
	Married, n (%)	232 (40.4)	494 (33.61)^h^	473 (34.20)^i^	423 (36.94)	315 (34.88)^i^	255 (31.99)^h^	533 (34.86)^i^
	≤ HS^j^ education, n (%)	121 (21.1)	454 (30.88)^g^	415 (30.00)^g^	330 (28.82)^g^	286 (31.67)^g^	266 (33.38)^g^	466 (30.48)^g^
	LGB^k^, n (%)	40 (6.9)	168 (11.43)^h^	159 (11.49)^h^	139 (12.14)^h^	115 (12.74)^g^	93 (11.67)^h^	175 (11.45)^h^
**Smoking variables**								
	FTND^l^, mean (SD)	5.4 (2.3)	5.8 (2.1)^g^	5.8 (2.2)^g^	5.8 (2.2)^g^	5.9 (2.1)^g^	5.9 (2.1)^g^	5.8 (2.2)^g^
	>20 cpd^m^, n (%)	174 (30.3)	520 (35.37)^i^	468 (33.84)	388 (33.89)	307 (33.99)	268 (33.62)	518 (33.88)
	Past month e-cigarette use, n (%)	179 (31.2)	532 (36.19)^i^	506 (36.59)^i^	413 (36.07)	323 (35.77)	287 (36.01)	553 (36.17)^i^
	CQSS^n^, mean (SD)	4.2 (0.7)	3.9 (0.8)^g^	3.9 (0.8)^g^	4.0 (0.8)^g^	4.0 (0.8)^g^	3.9 (0.8)^g^	3.9 (0.8)^g^
	AIS-sensations^o^, mean (SD)	3.1 (0.5)	2.9 (0.5)^g^	2.8 (0.4)^g^	2.8 (0.4)^g^	2.8 (0.4)^g^	2.8 (0.4)^g^	2.8 (0.4)^g^

^a^ADS: affective disorders and symptoms.

^b^PTSD: posttraumatic stress disorder.

^c^PD: panic disorder.

^d^GAD: generalized anxiety disorder.

^e^SAD: social anxiety disorder.

^f^2+ ADS: screened positive for 2 or more affective disorders.

^g^*P*<.001.

^h^*P*<.01.

^i^*P*<.05.

^j^HS: high school.

^k^LGB: identify as lesbian, gay, or bisexual.

^l^FTND: Fagerström Test for Nicotine Dependence.

^m^cpd: cigarettes per day.

^n^CQSS: Commitment to Quitting Smoking Scale.

^o^AIS-sensations: Avoidance and Inflexibility Scale–sensations subscale.

**Table 3:** Treatment acceptability (subjective and objective indices) by affective disorders and symptoms group.

Outcome variable			Comparison group (no ADS^a^)	Affective disorder symptomology subgroup					
				Depression	PTSD^b^	PD^b^	GAD^d^	SAD^e^	2+ ADS^f^
**Subjective, n/N (%)**									
	Satisfied with website		359/473 (75.89)	938/1123 (83.53)^g^	910/1090 (83.48)^g^	734/891 (82.38)^h^	598/699 (85.55)^i^	540/640 (84.38)^g^	991/1184 (83.69)^g^
	Website useful for quitting		334/483 (69.15)	835/1162 (71.86)	829/1126 (73.62)	664/918 (72.33)	527/714 (73.81)	487/654 (74.46)	892/1221 (73.05)
	Would recommend website to friend		369/409 (90.22)	957/1021 (93.73)	920/986 (93.31)	738/793 (93.06)	604/638 (94.67)^h^	537/575 (93.39)	1008/1079 (93.42)
**Objective**									
	Number of log-ins, mean (SD)		7.9 (19.9)	–^j^	–	–	5.8 (15.4)^i^	–	–
	**ACT^k^ website**								
		Mean (SD)	9.3 (21.7)	8.6 (31.2)	8.6 (31.5)	8.4 (26.0)	—	9.6 (40.6)	8.5 (30.7)
		n	270	733	690	561	—	389	759
	**Smokefree.gov**								
		Mean (SD)	6.7 (18.1)	4.6 (6.5)^i^	4.5 (6.2)^i^	4.5 (6.0)^i^	—^l^	4.5 (5.4)^i^	4.5 (6.3)^i^
		n	304	737	693	584	—	408	770
	Total minutes		35.4 (107.5)	29.2 (84.1)^i^	29.3 (86.4)^i^	26.9 (47.2)^i^	26.3 (70.3)^i^	31.4 (108.8)^i^	28.6 (82.7)^i^

^a^ADS: affective disorders and symptoms.

^b^PTSD: posttraumatic stress disorder.

^c^PD: panic disorder.

^d^GAD: generalized anxiety disorder.

^e^SAD: social anxiety disorder.

^f^2+ ADS: screened positive for 2 or more affective disorders.

^g^*P*<.01.

^h^*P*<.05.

^i^*P*<.001.

^j^Indicates significant treatment by subgroup interaction; results for these groups are separated by treatment arm in subsequent rows.

^k^ACT: acceptance and commitment therapy.

^l^Indicates no significant treatment by subgroup interaction.

### Cessation Outcomes

Cessation rates for each ADS group are shown in Table 4. The 12-month complete-case quit rate among participants without ADS was descriptively higher than all other groups (153/520, 29.4%). The quit rate was statistically lower for some but not all ADS subgroups. Specifically, participants in the depression (306/1267, 24.15%; odds ratio [OR] 0.78, 95% CI 0.62-0.98; *P*=.03), PTSD (294/1215, 24.19%; OR 0.78, 95% CI 0.62-0.98; *P*=.03), PD (229/1003, 22.83%; OR 0.73, 95% CI 0.57-0.92; *P*=.009), and two or more ADS (323/1332, 24.25%; OR 0.78, 95% CI 0.62-0.98; *P*=.03) groups exhibited statistically lower quit rates of 20% to 25% relative magnitude than the no ADS group; those who screened positive for GAD (195/792, 24.6%; *P*=.08) and SAD (176/696, 25.3%; *P*=.13) did not. None of the treatment arm by symptom group interactions were significant. Models examining the secondary cessation outcome where missing values were imputed as smoking resulted in a similar pattern of results, with the exception that the difference for the GAD subgroup became significant.

**Table 4:** Logistic regressions comparing 12-month cessation outcomes by affective disorders and symptoms group.

Characteristic		30-day PPA^a^, n/N (%)	Models controlling for trial stratification variables^b^ and treatment arm	
			OR^c^ (95% CI)	*P* value
**Complete case analyses**				
	No ADS^d^	153/520 (29.42)	reference group	reference group
	Depression	306/1267 (24.15)	0.78 (0.62-0.98)	.03
	PTSD^e^	294/1215 (24.19)	0.78 (0.62-0.98)	.03
	PD^f^	229/1003 (22.83)	0.73 (0.57-0.92)	.009
	GAD^g^	195/792 (24.62)	0.80 (0.62-1.03)	.08
	SAD^h^	176/696 (25.29)	0.82 (0.63-1.06)	.13
	2+ ADS^i^	323/1332 (24.25)	0.78 (0.62-0.98)	.03
**Missing=smoking analyses**				
	No ADS	153/574 (26.55)	reference group	reference group
	Depression	306/1470 (20.82)	0.74 (0.59-0.93)	.009
	PTSD	294/1383 (21.26)	0.76 (0.60-0.95)	.02
	PD	229/1145 (20.00)	0.70 (0.56-0.89)	.004
	GAD	195/903 (21.59)	0.77 (0.60-0.99)	.04
	SAD	176/797 (22.08)	0.79 (0.61-1.01)	.06
	2+ ADS	323/1529 (21.12)	0.75 (0.60-0.94)	.01

^a^PPA: point prevalence abstinence; no smoking, not even a puff in the last 30 days.

^b^Treatment stratification variables were gender, high school or less education, and smoking >20 cigarettes per day.

^c^OR: odds ratio.

^d^ADS: affective disorders and symptoms.

^e^PTSD: posttraumatic stress disorder.

^f^PD: panic disorder.

^g^GAD: generalized anxiety disorder.

^h^SAD: social anxiety disorder.

^i^2+ ADS: screened positive for 2 or more affective disorders.

### Post Hoc Analyses

To develop a better understanding of the factors contributing to lower quit rates among the symptom subgroups who were less likely to quit, post hoc analyses examined (1) whether each ADS group uniquely predicts abstinence after including baseline variables that differed between groups and were associated with cessation in the models and (2) whether website use (ie, log-ins, total time spent on website) mediated the relationship between ADS subgroup and abstinence.

As shown in Table 5, after adding the additional baseline variables to logistic regression models for each ADS subgroup, only screening positive for PD remained significantly negatively associated with cessation after controlling for baseline levels of nicotine dependence and commitment to quitting (OR 0.78, 95% CI 0.61-0.99; *P*=.04) in the analyses in which missing values were imputed as smoking. All other differences became nonsignificant and may represent variables that are part of a causal pathway between affective disorders and difficulty quitting. Mediation models tested the hypothesis that website use might indirectly explain the relationship between depression, PTSD, PD, and 2+ ADS subgroups (versus the no ADS group) and the primary cessation outcome [34]. However, none of the estimated indirect effects were significant, suggesting that fewer log-ins and less time spent on the websites did not help explain the observed lower quit rates for these ADS subgroups in this study (data not shown).

**Table 5:** Post hoc logistic regression analyses including additional covariates.

Characteristic		Models controlling for trial potential confounding variables		
		OR^a^ (95% CI)	*P* value	Additional covariates
**Complete case analyses**				
	No ADS^b^	reference group	reference group
	Depression	0.90 (0.71-1.13)	.36	FTND^c^, CQSS^d^
	PTSD^e^	0.90 (0.71-1.14)	.39	FTND, CQSS
	PD^f^	0.81 (0.63-1.03)	.09	FTND, CQSS
	2+ ADS^g^	0.87 (0.69-1.10)	.26	FTND, CQSS
**Missing=smoking analyses**				
	No ADS	reference group
	Depression	0.84 (0.67-1.06)	.15	FTND, CQSS
	PTSD	0.87 (0.69-1.10)	.24	FTND, CQSS
	PD	0.78 (0.61-0.99)	.04	FTND, CQSS
	2+ ADS	0.86 (0.67-1.11)	.24	CQSS

^a^OR: odds ratio.

^b^ADS: affective disorders and symptoms.

^c^FTND: Fagerström Test for Nicotine Dependence.

^d^CQSS: Commitment to Quitting Smoking Scale.

^e^PTSD: posttraumatic stress disorder.

^f^PD: panic disorder.

^g^2+ ADS: screened positive for 2 or more affective disorders.

## Discussion

### Principal Findings

To inform future treatment development efforts for smokers with ADS, we conducted the first study comparing treatment acceptability and long-term smoking cessation outcomes between smokers who screened positive for five common affective disorders (depression, PTSD, PD, GAD, SAD, as well as those who screened positive for ≥2 diagnostic categories). All ADS subgroups differed from the no ADS group (those who did not screen positive for any affective disorder) on most demographic and smoking characteristics assessed at baseline, similar to other reports [19,35,36].

All ADS subgroups had higher subjective acceptability ratings of their assigned website compared with the no ADS group. It may be that, relative to smokers without affective disorder symptomology, smokers with ADS see greater potential for Web-based smoking interventions or find the Web-based format particularly helpful and convenient, leading to greater subjective acceptability among those groups. Although unanticipated, this finding is important and demonstrates that smokers who screen positive for affective disorders are open to this treatment modality and find them helpful. However, the higher subjective ratings did not translate into greater use of the websites as one might expect. Although most ADS subgroups logged in significantly less to the Smokefree.gov website (with no differential log-in rates for the ACT-based website) relative to the no ADS group, all ADS subgroups spent significantly less time on both websites overall, which is on par with our hypotheses. The differential log-in rates for the websites across groups may mean that smokers with ADS engage more with Web-based cessation programs (ie, find them more acceptable) when they are grounded in ACT, presented in a stepwise fashion, or when they have features that prompt tracking.

Reasons for the discrepancy in the findings between the subjective and objective indices of acceptability are unclear. On the one hand, it may be that the cognitive and affective symptoms experienced by smokers with ADS impede them from using the websites as much as individuals without ADS. Alternatively, given that the websites in the parent trial were designed for the general population of smokers, it may also be that smokers with ADS found them less personally relevant, which could have contributed to them having lower levels of engagement [37-40].

The quit rates at 12-month follow-up for all symptom subgroups were promising (20% to 25%). However, in the primary analyses most symptom subgroups (those screening positive for depression, PTSD, PD, or ≥2 affective disorders) had significantly lower quit rates (23% to 24%) relative to the no ADS group (29%). Thus, there is some evidence of the possibility of differential outcomes being disorder-specific. However, all subgroups had descriptively lower quit rates than the no ADS group, suggesting that there is room to improve quit rates for all symptom subgroups and doing so would likely be significant at the population level considering the large proportion of smokers who screen positive for ADS.

Results from post hoc analyses suggest that the lower cessation rates among most groups may be influenced in part by their having greater levels of nicotine dependence and lower commitment to resist smoking in difficult situations at baseline. While greater levels of nicotine dependence among individuals with ADS is a robust finding in the literature [6,19,41], this is the first study to our knowledge demonstrating that these individuals also have significantly lower levels of commitment to resist smoking during difficult situations, which may be an important target to include in smoking interventions designed for these groups. However, unlike in previous studies of Web-based smoking interventions where greater use was associated with better cessation outcomes [42-45], the lower rates of cessation in the affective disorder subgroups were not mediated by website use. It may be that higher engagement only mediates cessation when the intervention adequately addresses the needs of the users. If the intervention does not address the user’s needs or is not perceived as relevant to the user, it follows that the user wouldn’t be as engaged and that engagement, per se, wouldn’t mediate cessation. Taken together, this suggests that increasing use alone among these groups may not be sufficient for improving cessation outcomes and that targeted interventions should also focus on addressing the unique, modifiable needs of smokers with affective conditions (eg, commitment to resist smoking in difficult situations) as a means of improving outcomes of these groups. Moreover, differences in nonmodifiable factors (eg, demographics) between these groups should also be considered when developing targeted interventions to increase their relevance for these groups. Future research is needed to determine if targeted Web-based interventions are associated with improved outcomes for these groups and if engagement mediates cessation outcomes in these cases.

### Strengths and Limitations

There are numerous strengths to this study including a large, geographically diverse sample of adult smokers living in the United States, the use of validated self-report instruments to assess mental health conditions, and assessing long-term cessation outcomes at 12-months with a high rate of outcome data retention. Despite these strengths, the limitations of this study should be considered when interpreting the results. The primary limitation is the exploratory nature of the study as secondary analyses embedded in a larger clinical trial [21]; thus, our conclusions should be considered preliminary. A second limitation to the study is the use of self-report screening instruments to assess ADS. Individuals who screened positive for affective disorders may not all have the disorders they screened positive for and instead have elevated, subthreshold symptoms of the disorders. In line with previous research indicating that even individuals with subthreshold symptoms of affective disorders are at risk for continued smoking [3,46] and that screening instruments for affective disorders can be used to predict cessation [2], this study highlights that the screening instruments used in this study can be useful in identifying smokers who are less likely to quit even without a formal diagnostic assessment. Finally, we did not biochemically verify self-reported smoking abstinence at the 12-month follow-up. However, biochemical validation of abstinence is considered unnecessary for population-level intervention studies that otherwise have no face-to-face contact with participants because requiring this can bias study results [32].

### Conclusions

This is the first study to compare treatment acceptability and long-term quit rates in response to Web-based smoking interventions between smokers who do versus do not screen positive for five common affective disorders. Findings suggest that while Web-based interventions are appealing to these groups, most individuals with ADS used their assigned website less and were less likely to quit smoking than their counterparts in response to the two interventions, each designed for the general population of smokers. Overall, these results support the need for developing targeted interventions for smokers with affective disorders and elevated affective symptomology.

## Abbreviations

ACT: acceptance and commitment therapy
AD: affective disorder
ADS: affective disorders and symptoms
AIS: Avoidance and Inflexibility Scale
CQSS: Commitment to Quitting Smoking Scale
FTND: Fagerström Test for Nicotine Dependence
GAD: generalized anxiety disorder
LGB: lesbian, gay, or bisexual
PD: panic disorder
PPA: point prevalence abstinence
PTSD: posttraumatic stress disorder
RCT: randomized controlled trial
SAD: social anxiety disorder
